# Random forest classification for predicting lifespan-extending chemical compounds

**DOI:** 10.1038/s41598-021-93070-6

**Published:** 2021-07-05

**Authors:** Sofia Kapsiani, Brendan J. Howlin

**Affiliations:** Department of Chemistry, FEPS, University of Surrey, Guildford, Surrey GU2 7XH UK

**Keywords:** Computational biology and bioinformatics, Drug discovery

## Abstract

Ageing is a major risk factor for many conditions including cancer, cardiovascular and neurodegenerative diseases. Pharmaceutical interventions that slow down ageing and delay the onset of age-related diseases are a growing research area. The aim of this study was to build a machine learning model based on the data of the DrugAge database to predict whether a chemical compound will extend the lifespan of *Caenorhabditis elegans*. Five predictive models were built using the random forest algorithm with molecular fingerprints and/or molecular descriptors as features. The best performing classifier, built using molecular descriptors, achieved an area under the curve score (AUC) of 0.815 for classifying the compounds in the test set. The features of the model were ranked using the Gini importance measure of the random forest algorithm. The top 30 features included descriptors related to atom and bond counts, topological and partial charge properties. The model was applied to predict the class of compounds in an external database, consisting of 1738 small-molecules. The chemical compounds of the screening database with a predictive probability of ≥ 0.80 for increasing the lifespan of *Caenorhabditis elegans* were broadly separated into (1) flavonoids, (2) fatty acids and conjugates, and (3) organooxygen compounds.

## Introduction

### Pharmacological interventions for longevity extension

Ageing is a major health, social and financial challenge, characterised by the deterioration of the physiological processes of an organism^1,2^. Ageing is a predominant risk factor for many conditions including various types of cancers, cardiovascular and neurodegenerative diseases^3,4^. Interventions targeting the cellular and molecular process of ageing have the potential to delay and protect against age-related conditions.

Several ageing studies have identified interventions that extend the lifespan of model organisms ranging from nematodes and fruit flies to rodents, using dietary restrictions, genetic modifications and pharmaceutical interventions. Lee et al*.* (2006) presented the first evidence that long-term dietary deprivation can improve longevity in a multicellular species, *Caenorhabditis elegans* (*C. elegans)*^5^. A few years later, Harrison et al*.* (2009) found that treating mice with rapamycin, an inhibitor of the mTOR pathway, extended the median and maxim lifespan of the mice^6^. Additionally, Selman et al*.* (2009) reported that genetic deletion of S6 protein kinase 1, a component of the mTOR signalling pathway, increased the lifespan of mice and protected against age-related conditions^7^.

Ye et al*.* (2014) developed a pharmacological network to identify pharmacological classes related to the ageing of *C. elegans*^8^. The network showed that resistance to oxidative stress and lifespan extension clustered in a few pharmacological classes, most of them related to intercellular signalling^8^. Moreover, Putin et al*.* (2016) developed a deep learning neural network that predicted human chronological age from a basic blood test^9^. The study identified the top five most critical blood markers for determining chronological age in humans, which were albumin, glucose, alkaline phosphatase, urea and erythrocytes^9^. Additionally, Mamoshina et al*.* (2018) developed a deep learning-based haematological ageing clock using blood samples from Canadian, South Korean, and Eastern European populations, with millions of subjects^10^. The findings showed that population-specific ageing clocks were more accurate in predicting chronological age and quantifying biological age than generic ageing clocks^10^.

Barardo et al*.* (2017) built a random forest model to predict whether a compound would increase the lifespan of *C. elegans* based on the data of the DrugAge database^1,4^. The features used to build the model were molecular descriptors and gene ontology terms. Feature selection was performed using random forest’s feature importance measure. The best performing model, with an AUC score of 0.80, was applied to predict the class of the compounds in the DGIdb database.

### Purpose of the work

This study builds on the work conducted by Barardo et al*.* (2017) to further explore the use of the DrugAge database for predicting compounds with anti-ageing properties^4^. Specifically, the random forest algorithm was employed to predict whether a compound will increase the lifespan of *C. elegans*. This was achieved by building five predictive models, each using different descriptor types, based on the data of the DrugAge database published by Barardo et al*.* (2017)^4^. The features of the models were molecular fingerprints and/or molecular descriptors calculated from the structure of the compounds in the DrugAge database. Feature selection was performed using variance and mutual information-based methods. To the best of our knowledge, this is the first implementation of molecular fingerprints for building machine learning models based on the entries of the DrugAge database. The best performing model was applied to predict the class of the compounds in an external database, consisting of 1738 small-molecules extracted from the DrugBank database^11^.

### Random forest models

Random forest is a supervised machine learning technique, consisted of an ensemble of decision trees, where each tree is trained independently using a random subset of the data^12^. The random forest model is widely used in chemo- and bioinformatics related tasks as it is robust to overfitting on high dimensional databases with a small sample sizes^4^.

The choice of chemical descriptors can significantly impact the quality and predictions of quantitative structure–activity relationship (QSAR) models. Descriptors represent chemical information of molecules in a digital or numerical way that is suitable for model development and are computer-interpretable^13,14^. In this study, 2D and 3D molecular descriptors were calculated using the Molecular Operating Environment (MOE) software^15^. 2D descriptors are calculated from the 2D structure of a molecule and provide information related to its structural, topological and physicochemical properties^16^. On the other hand, 3D descriptors are generated from the 3D structure of a chemical compound and include electronic parameters (e.g. dipole momentum), quantum–chemical descriptors (e.g. HOMO and LUMO energies), and surface:volume descriptors^14,17,18^.

Molecular fingerprints represent the molecule’s structure using binary vectors, where 1 corresponds to a particular feature or structural group being present and 0 that it is absent. Herein, extended-connectivity fingerprints (ECFP) of 1024- and 2048-bit lengths and RDKit topological fingerprints of 2048-bit length were generated in the RDKit Python environment^19^. Lastly, the combination of molecular descriptors with ECFPs was tested.

## Results and discussion

### Feature selection

Variance and mutual information filter-based methods were employed to select a subset of relevant features for predicting the anti-ageing properties of the molecules in the dataset. This was performed only for the training set which contained 80% of compounds in the dataset. Feature selection reduced the number of variables used by each model, making computational calculations less expensive. The median AUC scores and standard deviation of tenfold cross-validation (on the training set) obtained by random forest classification for each feature subset can be found in Supplementary Fig. 1, Additional File 1. The feature subset with the highest AUC score in 10-fold cross-validation was selected for classifying the compounds in the test set. In cases where two feature subsets achieved the same AUC score, the subset with the lowest standard deviation was used.

### Model selection

The test set contained 20% of the data not used in training the models. The performances of the random forest classifiers on the test and train set as well as the optimal number of variables obtained by feature selection are shown in Table 1.

**Table 1:** Performances on tenfold cross-validation and the test set achieved by each descriptor type; ECFP (1024-bit length), ECFP (2048-bit length), RDKit fingerprints (2048-bit length), molecular descriptors and the combination of ECFP (1024-bit length) with molecular descriptors.

Model	Number of selected features	Cross-validation on training set (AUC ± SD) (%)	Test set (AUC) (%)
ECFP_1024	55	79.4 ± 4.8	79.3
ECFP_2048	504	78.9 ± 4.2	77.6
RDKit5	654	83.6 ± 5.3	77.7
MD	69	82.3 ± 4.1	81.5
ECFP_1024_MD	33	82.8 ± 4.0	80.6

The number of features selected by applying variance and mutual information-based methods is reported.

**Figure 1:**
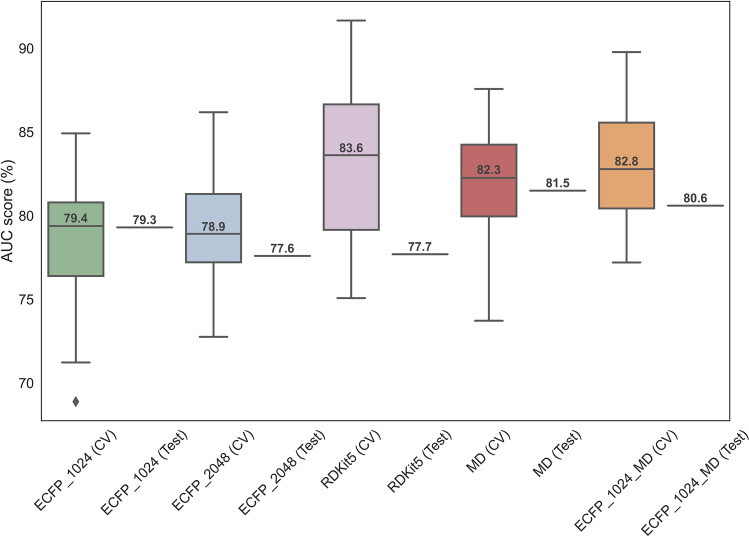
Box-and-whisker plot displaying the distribution of the AUC for the tenfold cross-validation (CV) on the training set and the AUC score for the single measurement taken on the test set, obtained by random forest classification. Each box represents the cross-validation data for a different model, where ECFP of 1024-bit length is shown in green, ECFP of 2048-bit length in blue, RDKit fingerprints in pink, molecular descriptors in red and the combination of ECFP of 1024-bit length with molecular descriptors are represented in orange colour. The value reported within the boxes is the median AUC value of the tenfold cross-validation. The points outside the boxplots represent possible outliers.

As illustrated in Fig. 1, the predictive performances of the random forest models built with ECFP and MD features did not significantly drop for classifying the compounds in the test set and were compatible with the spread of the AUC scores from cross-validation. This indicated that overfitting was minimised. On the other hand, the performance of the classifier using topological RDKit fingerprints on the test set dropped by approximately 6%. Therefore, the RDKit5 model was not selected for predicting the effect of the compounds in the screening database on the lifespan of *C. elegans*.

**Figure 2:**
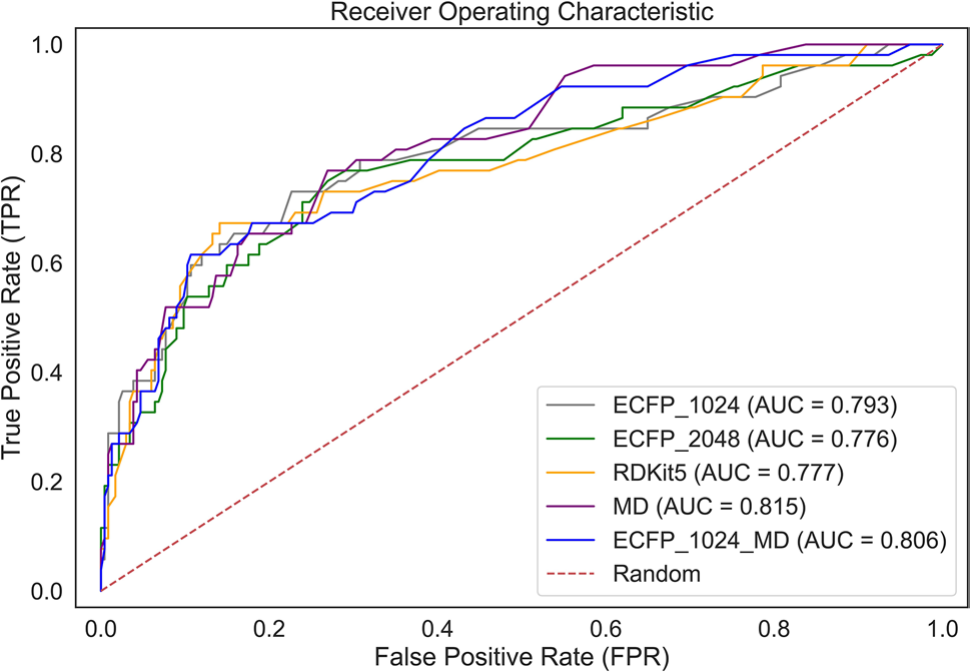
The ROC curves comparing the performances of the ECFP_1024 (in grey), ECFP_2048 (in green), RDKit5 (in orange), MD (in purple) and ECFP_1024_MD (in blue) for classifying the compounds in the test set. The AUC scores are reported for each descriptor type. The red dashed line corresponds to a random classifier, that gives random answers, with an AUC value of 0.5^20^.

The receiver operating characteristic (ROC) curve is the plot of the True Positive Rate (TPR) against the False Positive Rate (FPR) at varying classification thresholds. The ROC curves, displayed in Fig. 2, compare the performances of the descriptor types for classifying the samples of the test set. The figure illustrates that the five random forest models performed better than a random prediction.

The best performing model, selected by its ability to correctly classify the compounds in the test set, was used for predicting the class of the compounds in the screening dataset. The classifier built solely with molecular descriptors (MD), achieved the highest AUC score for predicting the class of the compounds in the test set. Combining MD with ECFP_1024, the feature type used to obtain the model with the second-highest predictive ability, did not further improve the performance of the classifier. The ECFP_1024 features could have provided additional information that was not useful to the random forest classifier making the predictions more difficult. Therefore, the MD model, which had an AUC score of 0.815 for classifying the compounds in the test set, was selected for further analysis.

### Confusion matrix

**Figure 3:**
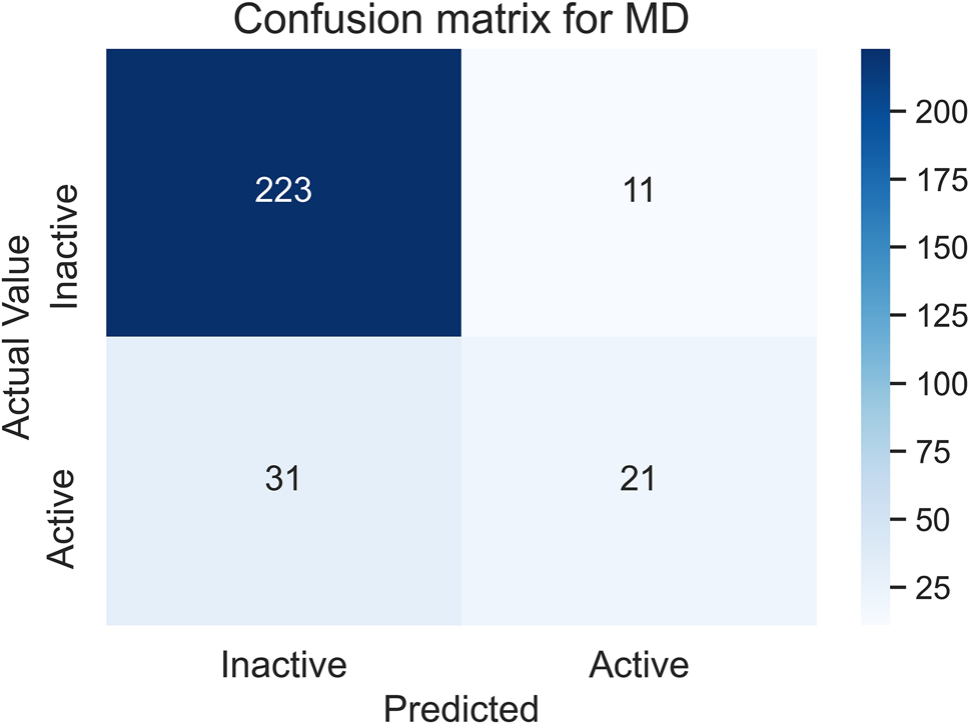
The confusion matrix was obtained from the performance of the MD model on the test set. The test set had a total of 286 compounds, of which 52 were active and 234 were inactive.

The confusion matrix of the MD model for predicting the class of the molecules in the test set is shown in Fig. 3. The classification accuracy of the model was 0.853 and the AUC score was 0.815.

The calculation of the Positive Predictive Value (PPV), Equation, and Negative Predictive Value (NPV), Equation, is shown below:

1 $$\begin{aligned} {\text{PPV}} & = {\text{~}}\frac{{{\text{True~}}\;{\text{Positives}}}}{{{\text{True}}\;{\text{~Positives}} + {\text{False}}\;{\text{~Positives}}}} \\ {\text{PPV}} & = ~\frac{{21}}{{21 + 11~}} = 65.6~\% \\ \end{aligned}$$

2 $$\begin{aligned} {\text{NPV}} & = ~\frac{{{\text{True}}\;{\text{~Negatives}}}}{{{\text{True}}\;{\text{~Negatives}} + {\text{False}}\;{\text{~Negatives}}}} \\ {\text{NPV}} & = ~\frac{{223}}{{223 + 31~}} = 87.8~\% \\ \end{aligned}$$

In binary classification, the PPV and NPV are the percentage of correctly classified compounds among all compounds predicted as positives or negatives, respectively. Herein, the PPV and NPV indicate that the random forest model performed better on correctly classifying inactive compounds than active ones. The data used in this study was imbalanced as approximately 79% of the samples were negative entries. Thus, a random prediction that a compound is inactive had a much higher initial probability of being correct. To handle the imbalanced data, the “class_weight” argument of the random forest algorithm was set to “balanced”, which penalises misclassification of the minority class (i.e. the positive samples)^21^. The remaining parameters of the random forest model were left to the default settings of the *scikit-learn* Python library (please refer to the “Random forest settings” section in the Methods)^22^. This increased the PPV for classifying the compounds in the test set from 61.1% (value without balancing the class weights) to 65.6% (score achieved after balancing the class weights), while the NPV was reduced by 0.2%.

### Feature importance

Investigating which features are considered “more important” by black-box models such as random forest can aid understanding of how these models make predictions. In this experiment, the feature relevance was measured using the “Gini importance” of the random forest algorithm. The selected model, MD, was composed of 69 molecular descriptors calculated by the MOE software^23^. The table containing the full feature ranking can be found in Additional File 2. The analysis was focused on the top 30 features with the highest Gini importance (Table 2), which contained both 2D and 3D molecular descriptors.

**Table 2:** Top 30 features ranked by Gini importance for the MD random forest model. The description of the features was taken from the MOE software documentation^23^.

Gini importance	Feature	Description
0.062	a_nN	Number of nitrogen atoms
0.029	PEOE_VSA + 2	Total positive van der Waals surface area of atoms with a partial charge in the range of 0.10 to 0.15
0.026	vsurf_D8	Hydrophobic volume
0.024	h_pKa	The pKa of the reaction that removes a proton
0.023	SMR_VSA6	Sum of van der Waals surface areas such that the molar refractivity contribution is in the range of 0.485 to 0.560
0.023	rsynth	A value in [0,1] indicating the synthetic reasonableness, or feasibility, of the chemical structure
0.022	PEOE_VSA-4	Total positive van der Waals surface area of atoms with a partial charge in the range of − 0.25 to − 0.20
0.021	PEOE_VSA + 4	Total positive van der Waals surface area of atoms with a partial charge in the range of 0.20 to 0.25
0.021	PEOE_VSA-6	Total positive van der Waals surface area of atoms with a partial charge that is less than − 0.30
0.021	PEOE_VSA_PPOS	Total positive van der Waals surface area of atoms with a partial charge that is greater than 0.20
0.020	chi0_C	Carbon connectivity index (order 0)
0.020	Q_VSA_PNEG	Total negative polar van der Waals surface area of atoms of with a partial charge that is less than − 0.20
0.020	PEOE_VSA_POL	Total polar van der Waals surface area of atoms of which the absolute value of their partial charge is greater than 0.20
0.020	chi0v_C	Carbon valence connectivity index (order 0)
0.019	SMR_VSA3	Sum of van der Waals surface areas such that the molar refractivity contribution is in the range of 0.35 to 0.39
0.019	Q_VSA_PPOS	Total positive van der Waals surface area of atoms with a partial charge that is greater than 0.20
0.018	b_single	Number of single bonds
0.018	a_count	Number of atoms
0.018	SlogP_VSA3	Sum of van der Waals surface areas such that the logP(o/w) is in the range of 0.0 to 0.1
0.018	PEOE_VSA_PNEG	Total negative polar van der Waals surface area of atoms of with a partial charge that is less than − 0.20
0.017	TPSA	Topological polar surface area
0.017	zagreb	Zagreb index
0.017	weinerPol	Wiener polarity number
0.017	opr_brigid	The number of rigid bonds
0.017	Kier3	Third kappa shape index
0.016	PEOE_VSA-1	Total positive van der Waals surface area of atoms with a partial charge in the range of − 0.10 to − 0.05
0.016	chi0	Atomic connectivity index (order 0)
0.016	Kier2	Second kappa shape index
0.016	SlogP_VSA2	Sum of van der Waals surface areas such that the logP(o/w) is in the range of − 0.2 to 0.0
0.015	a_nH	Number of hydrogen atoms

The highest-ranking features were broadly separated into the following categories (1) atom and bond counts (2) topological and (3) partial charge descriptors.

Atom and bond counts are simple descriptors that do not provide any information on molecular geometry or atom connectivity. The highest-ranking atom and bond count descriptors were a_nN, b_single, a_count, opr_brigid, and a_nH. While very simplistic, the atom and bond counts outperformed more complex 2D and 3D molecular descriptors. This is because atom and bond counts can partially capture the overall properties of a compound such as size, hydrogen bonding and polarity, which often impact the activity of a drug^24^. The number of nitrogen atoms, a_nN, was the top-ranking feature of the MD random forest model with a Gini importance score of 0.062. This is consistent with the results of Barardo et al*.* (2017) where a_nN was also ranked highest for predicting the class of the compounds in the DrugAge database^4^. Nitrogen atoms could have affected the physicochemical properties of the drugs as well as the interactions and binding of the molecules with target residues.

The highest-ranking topological descriptors included chi0_C, chi0v_C, zagreb, weinerPol, Kier3, chi0 and Kier2. Topological descriptors take into account atom connectivity. The descriptors are computed from molecular graphs, where atoms are represented by vertices and the bonds by edges^25^. These descriptors can provide information on the degree branching of the structure as well as molecular size and shape^25^. Although topological descriptors are extensively used in predictive modelling, they are usually hard to interpret^26^. Topological descriptors may have provided information on how well a molecule fits in the binding site and along with atom counts the interactions with the binding residues.

Top ranking partial charge descriptors were PEOE_VSA + 2, PEOE_VSA-4, PEOE_VSA + 4, PEOE_VSA-6, PEOE_VSA_PPOS, Q_VSA_PNEG, PEOE_VSA_POL, Q_VSA_PPOS and PEOE_VSA_PNEG. The “PEOE_” prefix denotes descriptors calculated using the partial equalization of orbital electronegativity (PEOE) algorithm for quantification of partial charges in the $\sigma$-system^27,28^. On the other hand, descriptors prefixed with “Q_” were calculated using the Amber10:EHT force field^23^. In a ligand-receptor system, partial charges can play a key role in the binding properties of the molecule as well as molecular recognition.

### Predicting potential lifespan-extending compounds

The MD random forest model was applied to predict the class compounds in an external database, consisting of 1738 small-molecules obtained from the DrugBank database^11^. The top-ranking compounds with a predictive probability of $\ge 0.80$ for increasing the lifespan of *C. elegans* are shown in Table 3. The full ranking of the molecules in the screening database can be found in Additional File 2. The compounds were broadly separated into the following categories; (1) flavonoids, (2) fatty acids and conjugates, and (3) organooxygen compounds. The compounds were classified based on categories “Class” and “Sub Class” of the chemical taxonomy section of the DrugBank database (provided by Classyfire) or assigned manually if not present^29^.

**Table 3:** Chemical compounds from the screening database with a predictive probability of 0.80 or above for increasing the of *C. elegans.*

Compound name	Predictive probability	Compound category
Diosmin	0.96	Flavonoids
Gamolenic acid	0.95	Fatty acids and conjugates
Rutin	0.95	Flavonoids
Hesperidin	0.94	Flavonoids
Lactose	0.89	Organooxygen compounds
6''-O-Malonyldaidzin	0.84	Flavonoids
Fidaxomicin	0.84	Other/Macrolide
Sucrose	0.83	Organooxygen compounds
Lactulose	0.83	Organooxygen compounds
Sodium aurothiomalate	0.82	Fatty acids and conjugates
Alloin	0.81	Other
Rifapentine	0.81	Other/Macrolactams
Plecanatide	0.80	Other/Polypeptides
Calcifediol	0.80	Other/Steroids and steroid derivatives
Chlortetracycline	0.80	Other/Tetracyclines

### Flavonoids

Flavonoids are a group of secondary metabolites in plants that are common polyphenols in the human diet^30^. Major nutritional sources include tea, soy, fruits, vegetables, wine and nuts^30,31^. Flavonoids are separated into subclasses based on their chemical structure, including flavones, flavonols, flavanones, and isoflavones^30^.

Flavonoids have been associated with health benefits for age-related conditions such as metabolic diseases, cancer, inflammation and cognitive decline^30,31^. Possible mechanisms of action include antioxidant activity, scavenging of radicals, central nervous system effects, alteration of the intestinal transport, sequestration and processing of fatty acids, PPAR activation and increase of insulin sensitivity^30^.

Diosmin was the top-hit molecule in the screening database, with a predictive probability of 0.96. Diosmin is a flavonol glycoside that is either extracted from plants such as Rutaceae or obtained synthetically^32^. It has anti-inflammatory, free radical scavenging, and anti-mutagenic properties and has been used medically to treat pain and bleeding of haemorrhoids, chronic venous disease and lymphedema^33^. Nevertheless, diosmin has poor aqueous solubility, which is a challenge for oral administration^34^. Kamel et al*.* (2017) found that a combination of diosmin with essential oils showed skin antioxidant, anti-ageing and sun-blocking effects on mice^34^. The underlying mechanisms for diosmin’s anti-ageing and photo-protective effects include enhancing lymphatic drainage, ameliorating capillary microcirculation inflammation and preventing leukocyte activation, trapping, and migration^34,35^.

**Figure 4:**
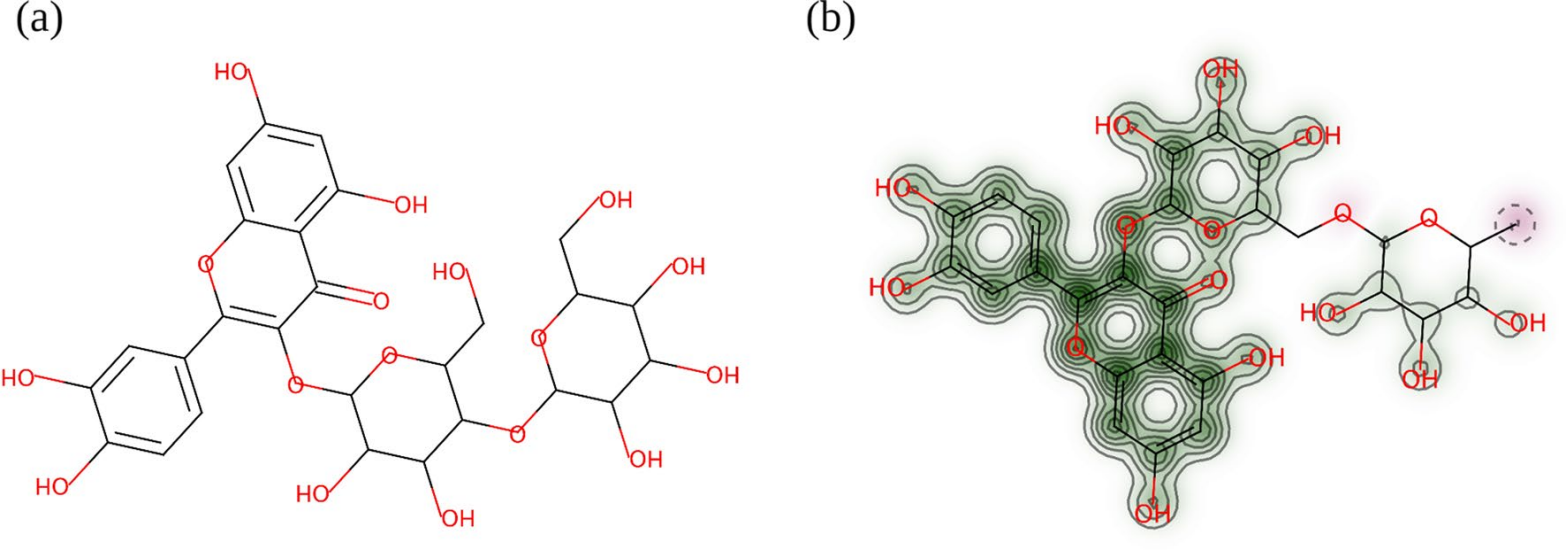
Similarity map for ECFP fingerprint with a default radius of 2 (**a**) structure of reference molecule Q3M from the DrugAge database (**b**) similarity map of rutin. In green colour are bits that if removed will decrease the similarity, whereas removing bits represented in pink colour will increase the similarity between the two compounds^38^. The figure was created in the RDKit Python environment^19^.

Other flavonoids that ranked high for increasing the lifespan of *C. elegans* were rutin and hesperidin with a predictive probability of 0.95 and 0.94, respectively. Rutin (or quercetin-3-rutinoside), is a flavonol glycoside that is abundant in many plants such as passionflower, apple, tea, buckwheat seeds and citrus fruits^36,37^. It possesses a range of biological properties including antioxidant, anticancer, neuroprotective, cardio-protective and skin-regenerative activities^36,37^. Rutin had a high structural similarity to other flavonoids in the DrugAge database and particularly with quercetin 3-O-β-d-glucopyranoside-(4 → 1)-β-d-glucopyranoside (Q3M). The Tanimoto coefficient between the RDKit fingerprints of Q3M and rutin was 0.99. The similarity map between the two compounds is shown in Fig. 4.

Q3M is a flavonoid abundant in onion peel that was found to extend the lifespan of *C. elegans*^39^. In the same study, even although rutin was found to improve the tolerance of *C. elegans* to oxidative stress, which is desirable for longevity, rutin (20 μg/mL) did not significantly affect the worm's lifespan^39^. On the other hand, a more recent study by Cordeiro et al*.* (2020) found that treatment of *C. elegans* with 15, 30 and 120 μM rutin increased the lifespan of the nematodes from 28 (control) to 30 days^40^.

Hesperidin has shown reactive oxygen species (ROS) inhibition and anti-ageing effects in the yeast species *Saccharomyces cerevisiae*^41^. Fernández-Bedmar et al*.* (2011) found that hesperidin extracted from orange juice had a positive influence on the lifespan of *D. melanogaster*^42^. Wang et al*.* (2020) showed that orange extracts, where hesperidin was the predominant phenolic compound, increased the mean lifespan of *C. elegans*^43^. In the same study, orange extracts were also found to promote longevity by enhancing motility and reducing the accumulation of age pigment and ROS levels^43^.

Soy isoflavones include genistein, glycitein, and daidzein. Genistein, a compound of the DrugAge, has been found to prolong the lifespan of *C. elegans* and increase its tolerance to oxidative stress^44^. Gutierrez-Zepeda et al*.* (2005) found that *C. elegans* fed with soy isoflavone glycitein had an improved resistance towards oxidative stress^45^. However, in comparison to control worms, the lifespan of *C. elegans* fed with glycitein (100 μg/ml) was not significantly affected^45^. The effect of daidzein (100 μM) on the lifespan of *C. elegans* in the presence of pathogenic bacteria was investigated by Fischer et al*.* (2012)^46^. The study found that daidzein had an estrogenic effect that extended the lifespan of the nematode in presence of pathogenic bacteria and heat^46^. Herein, we applied the MD random forest model to predict the effect of 6''-O-malonyldaidzin on the lifespan of *C. elegans.* 6''-O-Malonyldaidzin is an o-glycoside derivative of daidzein found in food products such as soybean, miso, soy milk and soy yoghurt^47^. Its predicted probability for extending the lifespan of the worm was 0.84.

### Fatty acids and conjugates

Lipid metabolism has an essential role in many biological processes of an organism. Lipids are used as energy storage in the form of triglycerides and can therefore aid survival under severe conditions^48^. Additionally, lipids have a key role in intercellular and intracellular signalling as well as organelle homeostasis^49^. Research on both invertebrates and mammals indicates that alterations in lipid levels and composition are associated with ageing and longevity^48,49^.

A recent review by Johnson and Stolzing (2019), on lipid metabolism and its role in ageing, summarised key lipid-related interventions that promote longevity in *C. elegans*^50^. Some of the studies presented in the review are reported here. O’Rourke et al*.* (2013), showed that supplementing *C. elegans* with the $\omega$-6 polyunsaturated fatty acids (PUFAs) arachidonic acid and di‐homo‐γ‐linoleic acid (DGLA) increased the worm’s starvation resistance and prolonged its lifespan by stimulating autophagy^51^. Similarly, Shemesh et al*.* (2017) found that DGLA extended the lifespan of *C. elegans* and maintained protein homeostasis in adulthood^52^. Additionally, Qi et al*.* (2017), found that treating *C. elegans* with $\omega$-3 PUFA $\alpha$-linolenic acid extended the nematodes lifespan^53^. The study indicated that the $\omega$-3 fatty acid underwent oxidation to generate a group of molecules known as oxylipins. The findings suggested that the increase of the worm’s lifespan could be a result of the combined effects of the α-linolenic acid and oxylipin metabolites^53^. Sugawara et al*.* (2013) found that a low dose of fish oils, which contained PUFAs eicosapentaenoic acid and docosahexaenoic acid, significantly increased the lifespan of *C. elegans*^54^*.* The authors proposed that a low dose of fish oils induces moderate oxidative stress that extended the lifespan of the organism. In contrast, large amounts of fish oils had a diminishing effect on the worm’s lifespan^54^.

Gamolenic acid or $~\gamma$-linolenic acid (GLA) was the second top-hit molecule of the screening database with a predictive probability of 0.95. GLA is an $\omega$-6 PUFA, composed of an 18-carbon chain with three double bonds in the 6th, 9th and 12th position^55^. Rich sources of GLA include evening primrose oil (EPO), black currant oil, and borage oil^56^. In mammals, GLA is synthesized from linoleic acid (dietary) via the action of the enzyme $\delta$-6 desaturase^55,56^. GLA is a precursor for other essential fatty acids such as arachidonic acid^55,56^. Conditions such as hypertension and diabetes as well as stress and various aspects of ageing, reduce the capacity of $\delta$-6 desaturase to convert linoleic acid to GLA^57^. This may lead to a deficiency of long-chain fatty acid derivatives and metabolites of GLA. GLA has been used as a constituent of anti-ageing supplements and has shown to possess various therapeutic effects in humans including improvement of age-related anomalies^55^.

Sodium aurothiomalate, with a lifespan increase probability of 0.82, is a thia short-chain fatty acid used for the treatment of rheumatoid arthritis and has potential antineoplastic activities^29,58^. In preclinical models, sodium aurothiomalate inhibited protein kinase C iota (PKCι) signalling, which is overexpressed in non-small cell lung, ovarian and pancreatic cancers^58^.

### Organooxygen compounds

Lactose, with a lifespan increase probability of 0.89, is a disaccharide found in milk and other dairy product. In the human intestine, lactose is hydrolysed to glucose and galactose by the enzyme lactase. Out of the compounds in the DrugAge database, lactose had the highest structural similarity with trehalose. Trehalose has been found to increase the mean lifespan of *C. elegans* by over 30%, without showing any side effects^59^. The Tanimoto coefficient between the RDKit fingerprint representations of trehalose and lactose was 0.85. Even though lactose has a high (Tanimoto) similarity to trehalose, Xing et al*.* (2019) found that lactose treatment at 10, 25, 50 and 100 mM concentrations shortened the lifespan of *C. elegans*^60^*.*

Sucrose, with a lifespan increase probability of 0.83, is a disaccharide composed of glucose and fructose^61^. It is used as the main form of transporting carbohydrates in fruits and vegetables^61^. Other sugars such as trehalose, galactose and fructose have been found to extend the lifespan of *C. elegans*^59,62,63^*.* Zheng et al*.* (2017) found the treating *C. elegans* with sucrose (55 μM, 111 μM, or 555 μM) had no significant effect on the organism’s mean lifespan^63^. On the other hand, a more recent study by Wang et al*.* (2020) found that treatment with 50 μM sucrose significantly increased the lifespan of the nematodes, while a concentration of 400 μM significantly shortened their lifespan^64^.

Lactulose, with a lifespan increase probability of 0.83, is a synthetic disaccharide composed of monosaccharides lactose and galactose^65^. Lactulose has shown to be an effective treatment for chronic constipation in elderly patients as well as improve the cognitive function in patients with hepatic encephalopathy^65,66^.

### Other classes of compounds

Other compounds with a predictive probability of ≥ 0.80 for increasing the lifespan of *C. elegans* included alloin, a constituent of *aloe vera* with a predictive probability of 0.81, as well as the antibiotics fidaxomicin (predictive probability = 0.84), rifapentine (predictive probability = 0.81) and chlortetracycline (predictive probability = 0.80).

Rifapentine is a macrolactam antibiotic approved for the treatment of tuberculosis^67^. Macrolactams are a small class of compounds that consist of cyclic amides having unsaturation or heteroatoms replacing one or more carbon atoms in the ring^29^. Other macrolactams such as rifampicin and rifamycin have been found to increase the lifespan of *C. elegans*^68^.

**Figure 5:**
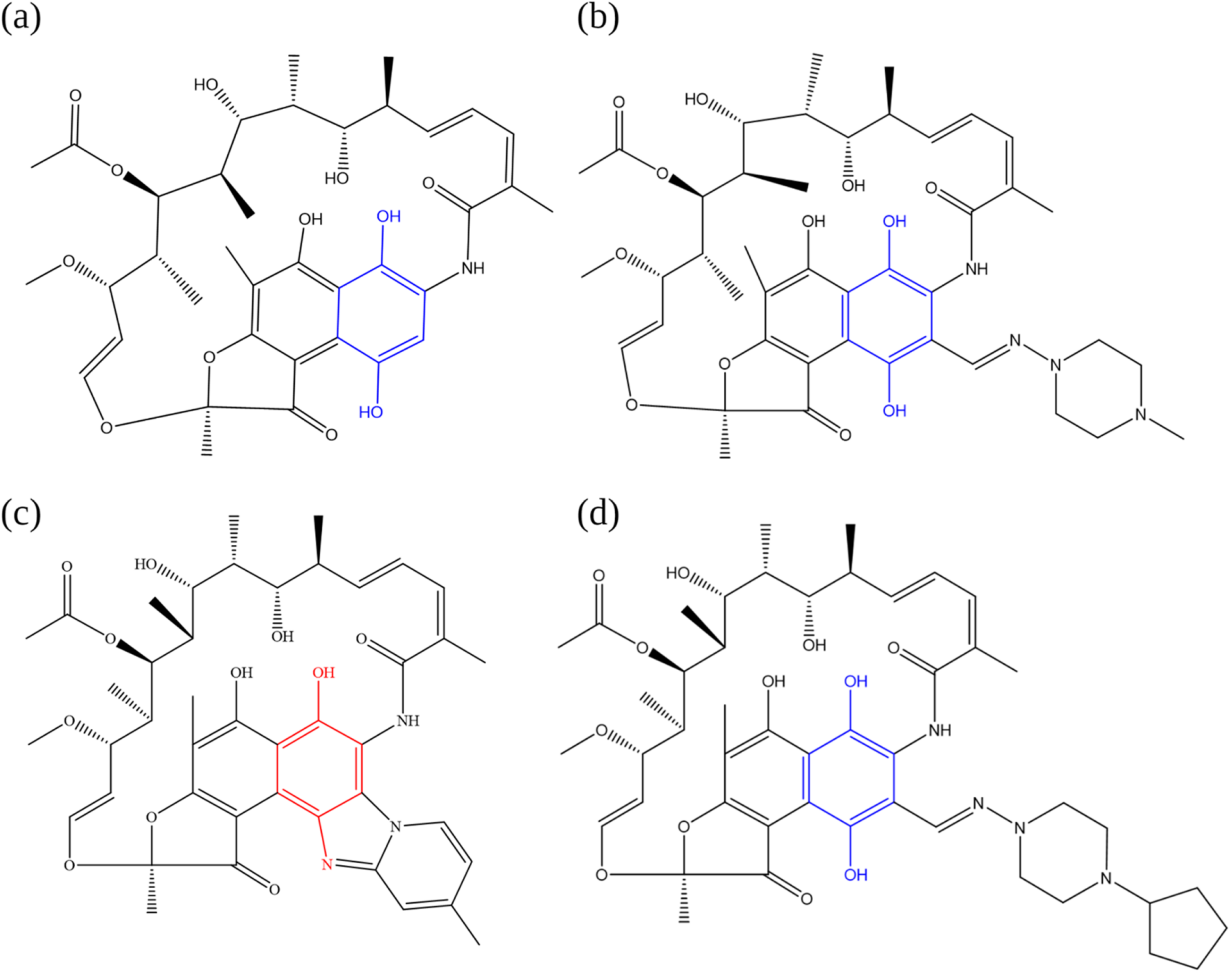
Chemical structure of (**a**) rifamycin SV (**b**) rifampicin (**c**) rifaximin and (**d**) rifapentine. The para-dihydroxynaphthyl moiety possessed by rifamycin SV, rifampicin and rifapentine is highlighted in blue. Rifaximin possesses a para-aminophenyl moiety incorporated in a ring system, highlighted in red^68^. The figure was designed in ChemDraw and redrawn from Golegaonkar et al*.* (2015)^68^.

Golegaonkar et al*.* (2015) showed that rifampicin reduced AGE products and extended the mean lifespan of *C. elegans* by 60%^68^. Advanced glycation end (AGE) products are formed from the non-enzymatic reaction of sugars, such as glucose, with proteins, lipids or nucleic acids^68^. AGE products have been implicated in ageing and age-related diseases such as diabetes, atherosclerosis, and neurodegenerative diseases^68^. The effect of two other macrolactams, rifamycin SV and rifaximin, on the lifespan of the nematode was also investigated. Rifamycin SV was found to exhibit similar activity to rifampicin, while rifaximin lacked anti-glycating activity and did not extend the lifespan of *C. elegans*. The authors suggested that the anti-glycation properties of rifampicin and rifamycin could be attributed to the presence of a para-dihydroxyl moiety, which was not present in rifaximin^68^. As shown in Fig. 5, this functional group is also present in rifapentine. Experimental testing would be required to investigate whether rifapentine possesses similar properties to rifampicin and rifamycin.

## Conclusions

Pharmaceutical interventions that modulate ageing-related genes and pathways are considered the most effective approach for combating human ageing and age-related diseases. Widely used strategies for identifying active compounds include screening existing drugs with potential anti-ageing properties.

In this study, the random forest algorithm was built to predict whether a compound would increase the lifespan of *C. elegans* using the entries of the DrugAge database, which contains molecules with known anti-ageing properties such as metformin, spermidine, bacitracin, and taxifolin. Our results provide an update on the findings of Barardo et al*.* (2017), by employing the latest version of the DrugAge database, which includes many more entries. Specifically, five random forest models were built using molecular fingerprints and/or molecular descriptors as features. Feature selection and dimensionality reduction were performed using variation and mutual information-based pre-selection methods. The best performing classifier, the MD model, was built using molecular descriptors and achieved an AUC score of 0.815 for classifying the compounds in the test set. Combining molecular descriptors with ECFPs did not further improve the model’s performance. The features of the MD model were ranked using random forest’s Gini importance measure. Among the 30 highest important features were molecular descriptors related to atom and bond counts, topological and partial charge properties.

The highest performing model was applied to predict the class of the compounds in the screening database which consisted of 1738 small-molecules from DrugBank. The compounds with a predictive probability of ≥ 0.80 for increasing the lifespan of *C. elegans* were broadly separated into (1) flavonoids, (2) fatty acids and conjugates, and (3) organooxygen compounds.

This study elucidated several molecules such as orange extracts, rutin, lactose and sucrose, that have been experimentally evaluated on *C. elegans* but were not entries of the predictive database. A limitation of our algorithm is that it does not consider the substance’s dose. For example, at certain concentrations sugars such as sucrose can promote longevity in *C. elegans*, whereas at higher concentrations such sugars have a detrimental effect on the lifespan of the nematodes^64^. Moreover, lactose, which received a predictive probability of 0.89 for increasing the lifespan of *C. elegans* by our model, was found to reduce the lifespan of *C. elegans* at 10–100 mM concentrations by Xing et al. (2019)^60^. Nevertheless, the compound could have a beneficial health effect at a different concentration.

Future work would involve in vivo testing of promising compounds such as $\gamma$–linolenic acid, rutin, lactulose and rifapentine to investigate their effect on the lifespan of *C. elegans,* as well as, reevaluate the effect of lactose at lower concentrations*.* Finally, further work would also explore how the predicted probability of lifespan increase is affected when testing two structurally similar compounds that promote longevity at different concentrations.

## Methods

### Dataset for predicting lifespan-extending compounds

The dataset published in the study by Barardo et al*.* (2017) contains positive entries, which are compounds that “increase the lifespan of *C. elegans*” and negative entries, compounds that “do not increase the lifespan of *C. elegans*”^4^. In particular, the dataset contains 1392 compounds of which 229 are positive and 1163 are negative entries^4^. The positive entries of this dataset were obtained from the DrugAge database of ageing-related drugs, (Build 2, release date: 01/09/2016), available on the Human Ageing Genomic Resources website^1,69^. DrugAge provides information on drugs, compounds and supplements with anti-ageing properties that have been found to extend the lifespan of model organisms^1^. The species include worms, mice and flies, with the majority of data representing *C. elegans*^4^. Data has been obtained from studies performed under standard conditions and contain information relevant to ageing, such as average/median lifespan, maximum lifespan, strain, dosage and gender where available^1^. The negative entries of the database used in the study of Barardo et al*.* (2017) were obtained from the literature.

At the time of writing, the latest version of the DrugAge database, Build 3 (release date: 19/07/2019), corrects for small errors and adds hundreds of new entries. Herein, the positive entries in the database used in Barardo et al*.* (2017) were replaced with the data from the newest version of DrugAge, Build 3. The same negative entries as Barardo et al*.* (2017) were used^4^. The modified database contained a total of 1558 compounds with 395 positive entries and 1163 negative ones. In this study, the term “DrugAge database” refers to the modified dataset with a total of 1558 compounds.

### Representation of chemical compounds

The chemical structures of the DrugAge dataset were converted into canonical SMILES strings using the Python package PubChemPy^70^. The SMILES strings were standardised by the Standardiser tool developed by Francis Atkinson in 2014^71^. Standardisation removed inorganic compounds, salt/solvent components and metal species as well as neutralised the compounds by adding or removing hydrogen atoms^71^. Stereoisomers, even if biologically may have different activities, were treated as duplicates as they had identical SMILES strings. For two or more stereoisomers in the same class, only one was kept. For duplicates in different classes, both were removed^72^. After standardisation and duplicate removal, the number of molecules in the DrugAge database was reduced to a total of 1430 compounds with 304 positive and 1126 negative entries. The predictive database used in this study can be found in Additional File 2.

### Molecular descriptor generation

The standardised SMILES strings were converted into mol files in the RDKit environment and opened in the MOE software^19,23^. The chemical structures were energy minimised in the Energy Minimize General mode of MOE using Amber10:EHT force field^23^. A total of 354 descriptors were calculated including all 2D, internal i3D and external x3D coordinate depended on 3D descriptors. Due to software limitation, few 3D descriptors ('AM1_E', 'AM1_Eele', 'AM1_HF', 'AM1_HOMO', 'AM1_IP', 'AM1_LUMO', 'MNDO_E', 'MNDO_Eele', 'MNDO_HF', 'MNDO_HOMO', 'MNDO_IP', 'MNDO_LUMO', 'PM3_E', 'PM3_Eele', 'PM3_HF', 'PM3_HOMO', 'PM3_IP', 'PM3_LUMO') could not be calculated for ten chemical structures. The missing values were replaced with the average value of the remaining chemical structures for the given descriptor.

### Molecular fingerprint generation

Molecular fingerprints were generated in the Python RDKit environment from the standardised SMILES strings^19^. ECFP of 1024-bits and 2048-bits length were calculated with an atomic radius of 2. These were represented as “ECFP_1024” and “ECFP_2048”, respectively. In addition to the ECFPs, RDKit topological fingerprints of 2048-bits length were generated with a maximum path length of 5 bonds and denoted as “RDKit5”.

Five random forest models were built using five different feature types and trained with the data of the DrugAge database. The feature types explored in this study, ECFP_1024, ECFP_2048, RDKit5, MD and ECFP_1024_MD, are summarised in Supplementary Table 1, Additional File 1. The ECFP_1024_MD feature was a combined descriptor type consisting of ECFPs of 1024 bit-length and molecular descriptors.

### Feature selection

Feature selection was implemented in the *scikit-learn* Python library^22^. Features with low variance were removed first, creating three feature subsets var_100, var_95 and var_90. The filters removed features with the same value in all entries (var_100), features that had greater than 95% of constant values (var_95) and features with more than 90% constant values, respectively (var_90)^73^.

Mutual Information (MI) is a filter-based feature selection method used to measure the dependency between two variables. The MI between variables $X$ and $Y$ is given by (Eq. 3):

3 $$MI\left( {X;Y} \right) = ~\mathop \sum \limits_{x} \mathop \sum \limits_{y} p\left( {x,y} \right)~log\frac{{p\left( {x,y} \right)}}{{p\left( x \right)p\left( y \right)}}$$

where $p\left( {x,y} \right)$ is the joint probability mass function and $p\left( x \right)$ and $p\left( y \right)$ are the marginal probability mass functions for $X$ and $Y$, respectively^74^. Herein, Adjusted Mutual Information (AMI) was calculated between each feature and the class labels. AMI was used as it is a variation of MI that adjusts for chance^75^.

For each of the feature subset, AMI was applied using the “adjusted_mutual_info_score” function of *scikit-learn* to order the features based on their AMI score^22^. The following settings were tested: using 5%, 10%, 25%, 50%, 75% and 100% of the features with the highest AMI score^73^. For example, if var_100 for MD contained 349 features, the database with 5% of the features would consist only of the 17 highest-ranking features. This process is outlined in Supplementary Fig. 2, Additional File 1.

### Tenfold cross-validation

Cross-validation was performed in the *scikit-learn* Python library using the “cross_val_score” function^22^. The predictive database was randomly split into 80% training and 20% test set. The tenfold cross-validation was performed only on the training set. The performance of the models was evaluated using the AUC measure. Cross-validation was repeated 10 times, yielding 10 AUC scores. The predictive accuracy reported was the median AUC value of the 10 measurements obtained by cross-validation. The median, rather than average, AUC score was calculated as the former is more robust to outliers^4^.

### Random forest settings

The random forest classifiers were built in the *scikit-learn* Python module^22^. To handle the unbalanced data used in this study, the random forest parameter “class_weight” was set to “balanced”. The remaining parameters of the random forest classifier were set to their default settings. The models were run with 100 estimators (number of trees in the forest) and the maximum number of features considered in each tree node was the square root of the total number of features. The AUC scores were calculated with the “roc_auc_score” matrix of *scikit-learn* using the “predict_proba” method^22^.

### Screening database

The best performing model on the test set was applied to predict the class of the compounds in an external database, where the effect of the compounds on the lifespan of *C. elegans* was mostly unknown. The external database consisted of small-molecules obtained from the External Drug Links database of DrugBank (version 5.1.5, released on 2020-01-03)^11^. The External Drug Links database contained a list of drugs and links to other databases, such as PubChem and UniProt, providing information on these compounds^11,76,77^.

Generation of SMILES strings, standardisation and descriptor calculation was performed in the same method used for the training (DrugAge) database, described in the above sections. Some of the entries of the DrugBank database were substances composed of more than one molecule, such as vegetable oils. These entries were either removed from the database or replaced by one of their main active ingredients. For example, “borage oil” was replaced with “gamolenic acid”. In the case of “soy isoflavones”, the major soy isoflavones (genistein, glycitein, and daidzein) had already been experimentally evaluated on the lifespan of *C. elegans*. Therefore, the entry was replaced with “6''-O-malonyldaidzin”, a derivative of daidzein with unknown activity. Stereoisomers were treated as duplicates and only one of them was kept. Substances and stereoisomers present in both the DrugBank and DrugAge databases were removed from the screening database. The resulting database consisted of a total of 1738 small-molecules.

### Tanimoto coefficient and similarity maps

The Tanimoto coefficients and similarity maps were computed in the Python RDKit environment^19^. The Tanimoto similarity is calculated between a reference molecule, which is known to be active, and a compound of interest with unknown activity.

Herein, the reference molecules were the positive entries of the DrugAge database. The compound with unknown activity was a selected entry from the screening database. The Tanimoto coefficient between the compound of interest with each of the reference molecules was calculated. The highest score achieved and the reference molecule used to obtain that score was reported. The Tanimoto coefficients were computed using the RDKit fingerprint representations of the compounds. Similarity maps were generated using ECFP fingerprint representations.

## Supplementary Information

Supplementary Information 1.

Supplementary Information 2.
