# Supplementary material for: Random forest classification for predicting lifespan-extending chemical compounds
Source: Sci Rep. 2021 Jul 5;11:13812. doi: 10.1038/s41598-021-93070-6 (PMC8257600; doi:10.1038/s41598-021-93070-6)
Supplement: Supplementary file 1 — Supplementary Information 1. [file 41598_2021_93070_MOESM1_ESM.docx]

**Appendix**

**Title page**

**Random forest classification for predicting lifespan-extending chemical compounds**
Sofia Kapsiani^1^, Brendan J. Howlin^1,*^

^1^Department of Chemistry, FEPS, University of Surrey, Guildford, Surrey, GU2 7XH, UK

*Corresponding author’s e-mail: b.howlin@surrey.ac.uk

**Supplementary Figure 1**


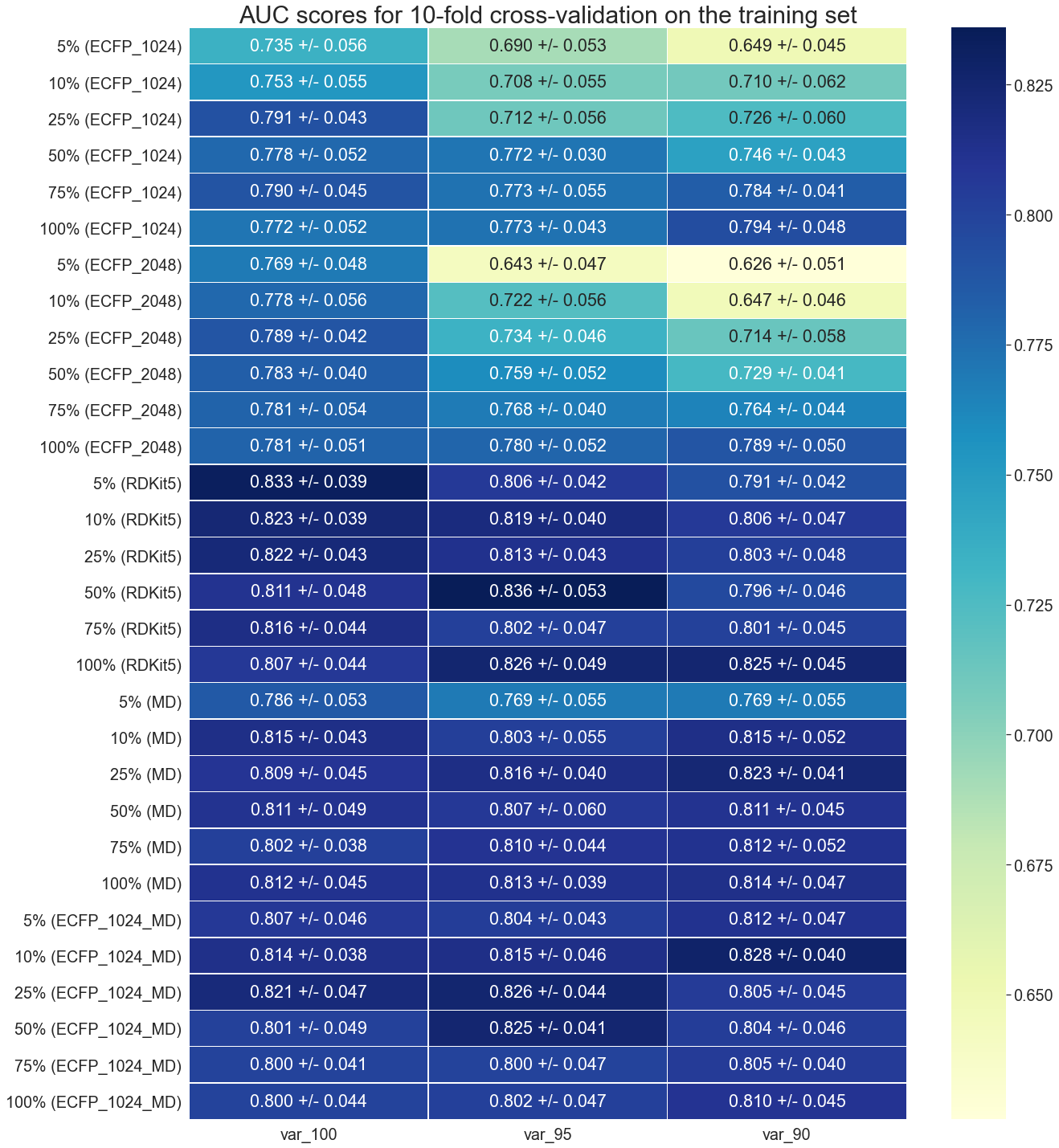


The median AUC scores and standard deviation of 10-fold cross-validation obtained by random forest classification. The predictive scores for different feature types, ECFP (1024-bit length), ECFP (2048-bit length), RDKit fingerprints, molecular descriptors and combination of ECFPs with molecular descriptors, are shown. The feature subset with the highest AUC score for each descriptor type is shown in bold. In cases where two feature subsets achieved the same AUC score, the subset that had the smallest standard deviation was used. The columns of the figure, var_100, var_95 and var_90 represent the variance thresholds, while the rows show the feature type and percentage of the top features (based on mutual information) selected in each case for the calculation of the AUC score. The variance thresholds var_95 and var_90 in combination with high mutual information thresholds significantly reduced the number of available features in the ECFP models, thereby, lowering the model’s predictive ability.

**Supplementary Table 1**

| **Database name** | **Feature description** | **Number of features** |
| --- | --- | --- |
| ECFP_1024 | ECFP of 1,024-bit length generated in the Python RDKit environment | 1,024 |
| ECFP_2048 | ECFP of 2,048-bit length generated in the Python RDKit environment | 2,048 |
| RDKit5 | RDKit topological fingerprints with a maximum path length of 5 bonds generated in the Python RDKit environment | 2,048 |
| MD | 2D and 3D molecular descriptors calculated in MOE™ | 354 |
| ECFP_1024_MD | Combination of “ECFP_1024” and “MD” descriptors | 1,378 |

Description of feature types explored in this study.

**Supplementary Figure 2**

Overview of the modelling process for feature selection and model evaluation. This procedure was followed for each feature type (ECFP_1024, ECFP_2048, RDKit5, MD and ECFP_1028_MD). The figure was generated in ChemDraw.
